# Author Correction: The economic commitment of climate change

**DOI:** 10.1038/s41586-024-07732-2

**Published:** 2024-06-24

**Authors:** Maximilian Kotz, Anders Levermann, Leonie Wenz

**Affiliations:** 1 Research Domain IV, Potsdam Institute for Climate Impact Research, Potsdam, Germany; 2 Institute of Physics, Potsdam University, Potsdam, Germany; 3 Mercator Research Institute on Global Commons and Climate Change, Berlin, Germany

**Keywords:** Projection and prediction, Interdisciplinary studies, Economics, Environmental health, Environmental economics

Correction to: *Nature* 10.1038/s41586-024-07219-0 Published online 17 April 2024

In the version of the article initially published, values in every second row in Extended Data Table 2 and Supplementary Tables 2–4 were wrongly printed as a decimal, rather than a percentage point. These values have been updated online. Ref. 21 was missing an author and has also been updated. The authors apologise for any confusion caused. These corrections do not affect any of the results of the manuscript.

